# New Appliances and Things Medical

**Published:** 1900-03-31

**Authors:** 


### NEW APPLIANCES AND THINGS MEDICAL.

[We shall be glad to receive, at our Office, 28 & 29, Southampton Street, Strand, London, W.C., from the manufacturers, specimens of all new preparations and appliances which may be brought out from time to time.]

### "PETANELLE" WOOL SURGICAL DRESSING AND OTHER PRODUCTS.

(Paté, Burke, and Co., 6, Wool Exchange, London, E.C.)

"Petanelle" is a name given to the substances produced by this company from peat, the particular article with which we are more especially concerned at the present moment being the "petanelle" surgical dressing. This is a soft, brown fibre, not so "short" as wood wool, nor quite so soft as cotton wool, but more elastic than either, while quite soft enough to make a comfortable dressing. It is very absorbent, and in one point seems to be superior to almost every material which we have employed for dressing purposes, namely, that its elasticity and resiliency are but very little impaired by wetting. In this respect it is very markedly superior to all preparations of cotton wool. For all purposes of splint padding this resiliency makes it especially valuable, and as a dressing it will also be found very useful, especially where any considerable quantity of discharge is expected. It is sold in bulk, and also made up in sheets enclosed between pieces of very open gauze, to take the place of Gamgee tissue. On the whole this seems to us to be the most generally useful form, as it can be cut to any size by a pair of scissors, and these "pads" can then be wetted with lotion if required without losing their shape. "Petanelle" wool is employed to make ladies' "towelines," for which purpose its elasticity and absorbent properties are very useful. "Petanelle" is also manipulated in such a manner as to be made into various fabrics, which, in proportion as the peat basis enters into their composition, retain its useful properties; and, finally, a "petanelle" disinfecting powder is made which, if freely used, is likely to be effectual in preventing smells. Altogether, a very useful series of products, out of which we select the "surgical wool," the "towelines," and the "accouchement sheets" as especially worthy of commendation

